# The association between shift work and possible obstructive sleep apnea: a systematic review and meta-analysis

**DOI:** 10.1007/s00420-021-01675-1

**Published:** 2021-03-07

**Authors:** Chen-Cheng Yang, Kuo-Wei Lee, Kazuhiro Watanabe, Norito Kawakami

**Affiliations:** 1 Department of Occupational and Environmental Medicine, Kaohsiung Municipal Siaogang Hospital, No. 482, Shanming Road, Siaogang District, Kaohsiung City, Taiwan; 2 Graduate Institute of Medicine, College of Medicine, Kaohsiung Medical University, Kaohsiung, Taiwan; 3 Department of Family Medicine, Kaohsiung Municipal Siaogang Hospital, Kaohsiung, Taiwan; 4 Department of Occupational and Environmental Medicine, Kaohsiung Medical University Hospital, Kaohsiung, Taiwan; 5 Department of Mental Health, Graduate School of Medicine, The University of Tokyo, Tokyo, Japan; 6 Environmental and Occupational Medicine Center, Kaohsiung Municipal Siaogang Hospital, Kaohsiung, Taiwan; 7 Department of Neurology, Kaohsiung Municipal Siaogang Hospital, Kaohsiung, Taiwan

**Keywords:** Shift work, Obstructive sleep apnea, Meta-analysis

## Abstract

**Background:**

Shift work is a workschedule, since industrial era and some employees work in shift. It causes a desynchronization of the biological clock with consequences on sleep amount and quality, such as insomnia and easy fatigue. Obstructive sleep apnea (OSA) is one of the sleep problems that are getting more and more attention, but studies on the association between shift work and OSA were rare. Herein, we aimed to conduct a systematic review and meta-analysis to investigate the association between shift work and possible OSA.

**Methods:**

This study was conducted according to Preferred Reporting Items for Systematic Reviews and Meta-Analyses guidelines. We queried PubMed, Embase, and Web of Science databases using a related set of keywords. The inclusion criteria were as follows: (1) participants were adult employees hired by a company or organization; (2) exposure was shift work; and (3) outcome was possible OSA according to examination or assessment.

**Results:**

We included six studies in the systematic review and five studies were selected for further meta-analysis. A random-effects model showed an association of shift work with a small, non-significant increase in possible OSA cases (pooled prevalence relative risk = 1.05; 95% CI 0.85–1.30; *p* = 0.65). This association occurred in both healthcare and non-healthcare workers group.

**Conclusion:**

The association between shift work and possible OSA remains inconclusive and could be small if not negligible. Future studies should assess the association between specific work schedules and specific OSA definitions.

**Trial registration number:**

PROSPERO ID: CRD42020156837

**Supplementary Information:**

The online version contains supplementary material available at 10.1007/s00420-021-01675-1.

## Introduction

### Obstructive sleep apnea

Obstructive sleep apnea (OSA) is a disorder involving breathing pauses frequently during sleep (Franklin and Lindberg 2015). According to the international classification of sleep disorders, 3rd ed. (ICSD-3) (AASM 2014), in adults without associated symptoms or comorbid disorders it was diagnosed when there are 15 or more primarily obstructive respiratory events per hour. In those with signs/symptoms or associated medical or psychiatric disorders the diagnosis of OSA was made when there are five or more predominantly obstructive respiratory events per hour (Sateia 2014). Common signs and symptoms of OSA include snoring or gasping during sleep and sleepiness or feel tired during the daytime (Franklin and Lindberg 2015; Park et al. 2011). However, patients with OSA are often unaware of the disease and are often notified by family members. The estimated OSA prevalence rate in the recent study was around 14% in men and 5% in women those age among 30–70 years, and the prevalence may increase to 20–30% in elder age or obesity population (Peppard et al. 2013). Previous studies have reported an association of OSA with atherosclerosis (Drager et al. 2011), cardiovascular events (Voulgaris et al. 2019), venous thromboembolism (Alonso-Fernandez et al. 2019), stroke (Redline et al. 2010), atrial fibrillation (Kendzerska et al. 2018), and sudden cardiac death (Gami et al. 2013). Moreover, previous studies have shown that OSA has increased health-related and social transfer costs, increased unemployment rates, and decreased income (Jennum et al. 2014; Jennum and Kjellberg 2011).

The risk factors for OSA include obesity (Ahlin et al. 2019; Peppard et al. 2000), family history of sleep apnea (Redline et al. 1995), and allergy (Calais et al. 2016; Jiang et al. 2016). There have been a few studies on work-related factors that affect OSA. Studies on occupational differences in the OSA prevalence (Kales and Straubel 2014; Schwartz et al. 2017) have reported that OSA is more prevalent among commercial drivers. Furthermore, a high OSA risk was observed among rescue and recovery workers after a huge explosion event (Webber et al. 2011). On the other hand, a study reported no significant association of solvent exposure with OSA (Schwartz et al. 2017). Nakata et al. and Tripathi et al. suggested that occupational stress could be a possible risk factor for OSA (Nakata et al. 2007; Tripathi et al. 2018).

### Shift work

There are comprehensively varying shift work schedules. The International Labour Organization defines shift work as a “a method of organization of working time in which workers succeed one another at the workplace”(International Labour Organization). In clinical situation, shift work is typically considered as “work beyond the typical daily working hours (around 7–8 a.m. to 5–6 p.m.)”. These include evening shift, night shift, graveyard shift, rotational shift, etc. (Costa 2003; Leso et al. 2020; Rosa and Colligan 1997; Straif et al. 2007). More than one of five shift workers suffered from insomnia(Drake et al. 2004; Pepin et al. 2018), and one of ten suffered from shift work sleep disorder (Drake et al. 2004). Moreover, shift work is associated with obesity (Biggi et al. 2008; Karlsson et al. 2001; Ramin et al. 2015); therefore, it might be associated with an increased OSA risk. Shift work disrupts the circadian rhythm and is associated with several health problems, including cardiovascular diseases (Torquati et al. 2018), diabetes mellitus (Morikawa et al. 2005; Pan et al. 2011), and poor mental health (Torquati et al. 2019). Circadian rhythm disruptions and related chronic conditions could facilitate OSA development. Furthermore, irregular work schedules could induce an inflammatory response (Amano et al. 2018), which might trigger OSA occurrence (Tripathi et al. 2018). Previous studies on the longitudinal association between shift work and OSA: have reported inconsistent findings (Joorabbaf et al. 2017; Seyedmehdi et al. 2016; Soylu et al. 2014; Walia et al. 2012).

Therefore, there is a need for a systematic review and meta-analysis to analyze and integrate the current evidence on the association between shift work and sleep apnea. This could contribute toward the understanding of the mechanisms of OSA development and provide evidence for occupational health professionals to consider in the prevention of shift work-related health problems.

### Purpose

Because the limited literatures on the association between shift work and clinically diagnosed OSA, we included mild symptoms/signs as an indicator of higher OSA risk in the review. We aimed to assess the association of shift work with a higher OSA risk. To our knowledge, this is the first systematic review and meta-analysis of this association. Given the ethical issues in conducting trials with shift work as the exposure, we evaluated current studies to investigate this association. We hypothesized that shift work was associated with a high OSA risk.

## Materials and methods

### Protocol and registration

This review was conducted according to the preferred reporting items for systematic reviews and meta-analyses (PRISMA) guidelines. This review protocol was registered at PROSPERO (ID. CRD42020156837), link websites: https://www.crd.york.ac.uk/prospero/display_record.php?RecordID=156837.

### Study selection

#### Data sources and search terms

We queried MEDLINE (PubMed), Embase, and Web of Science databases on 21st October 2019 for related studies. We do not limit the dates of studies published in these databases and all studies including these target keywords were considered. Two researchers (CCY and NK) performed a preliminary search using different key words. The researchers separately proposed a set of key search words that were subsequently as follows: working hour × [Title/Abstract] OR working time[Title/Abstract] OR day-time[Title/Abstract] OR night-time[Title/Abstract] OR shift work × [Title/Abstract] OR work shift × [Title/Abstract] OR temporary work[Title/Abstract] OR full-time[Title/Abstract] OR part-time[Title/Abstract] AND (sleep apnea syndromes) AND (longitudinal OR prospective OR cohort OR [follow AND up] OR observational). The search methods for Embase and Web of Science databases were modified as appropriate.

### Eligibility criteria

The eligibility criteria for study inclusion were as below: (1) adult employees work in a factory/company/organization; (2) exposure to shift work/non shift work; and (3) outcome was possible OSA according to examination or assessment.

### Process of article selection

Initially, two researchers (CCY and KWL) independently evaluated the titles and abstracts of the initially identified studies (the first screening). Subsequently, a comprehensive full-article screening was performed (the second screening) of studies which met the inclusion criteria and those with uncertain eligibility upon screening of the title and abstract. If Yang and Lee disagreed on the eligibility of a study, four researchers (CCY, KWL, KW, and NK) thoroughly assessed it to decide whether it was to be included or eliminated.

### Data collection

From each eligible study, we extracted information regarding the study characteristics, shift work, possible OSA cases, and the association between shift work and possible OSA. We contacted the authors of the inclusion study for further explanation if the study failed to or imprecisely reported the required data.

### Study characteristics

We obtained the following data regarding study characteristics: publication year, the country where the study was completed, sample size, sampling framework (clinical- or workplace-based), participant’s characteristics, number of outcome events (i.e., the number of participants with possible OSA) where appropriate.

### Shift work

We defined shift work as “work beyond regular working daytime hours”, including evening shift, night shift, fixed shift, on-call shift or rotating shift (Costa 2003; Leso et al. 2020; Rosa and Colligan 1997; Straif et al. 2007).

### Possible OSA cases

The outcomes were as follows: polysomnography diagnosis of OSA or questionnaires for OSA risk assessment. High risk was defined according to individual articles.

### Statistical analysis

We calculated all pool prevalence relative risks (RRs) from the number of possible OSA cases among the shift and non-shift workers. We estimated the standard error (SE) for the relative risks according to the 95% confidence interval (CI) for the relative risks. In this meta-analysis, we used the prevalence RR and its SE. Regarding the main analysis, the pooled prevalence RR with its 95% CI were calculated by means of meta-analysis of random-effects model. We also applied a random-effects model to analyze the possibility of heterogeneity in relative risks among these studies derived from their characteristics, including participants numbers and countries (Hunter and Schmidt 2000). Moreover, we used a fixed-effect model to conduct the meta-analysis. Among-study heterogeneity was analyzed using I2. We assessed publication bias using a funnel plot and performed the Egger’s test. We performed separate subgroup meta-analysis of the healthcare workers (HCW) and non-healthcare workers (non-HCW). All the analyses were performed using Review Manager Version 5.3 and R version 3.6.2.

## Results

### Selected studies

**Fig. 1:**
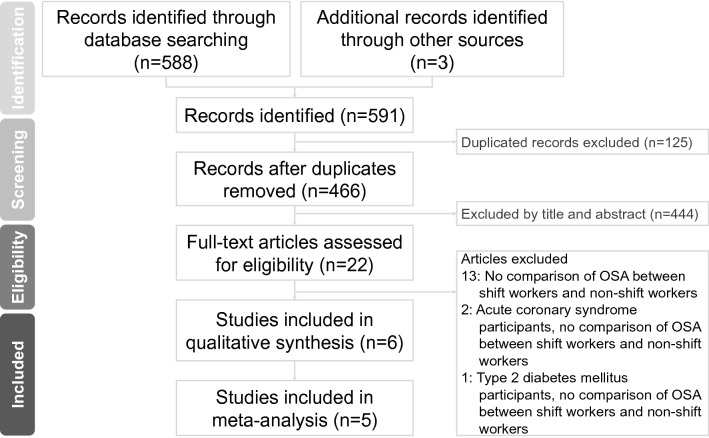
PRISMA flow diagram

Figure 1 shows the research selection process using a PRISMA flow diagram. The initial database search (PubMed, Embase, and Web of Science) identified 588 articles. In addition, screening of the references led to the inclusion of three additional studies (Seyedmehdi et al. 2016; Soylu et al. 2014; Yazdi et al. 2014). Subsequently, 125 duplicates were removed. After screening the titles and abstracts of the 466 studies left, two researchers (CCY and KWL) found 22 studies. After the subsequent full-article evaluating of the 22 studies, 16 articles were excluded for not meeting the following criteria: no OSA comparison between shift workers and non-shift workers (*N* = 13) or inclusion of participants with special diseases (*N* = 3). Finally, we included six studies in the qualitative review and five studies for further analysis.

### Study characteristics

Table 1 shows the six studies that met our inclusion criteria (Aydin Guclu et al. 2019; Joorabbaf et al. 2017; Seyedmehdi et al. 2016; Soylu et al. 2014; Walia et al. 2012; Yazdi et al. 2014). Among them, five were cross-sectional studies as follows: Yazdi et al. (225 and 245 shift and non-shift textile factory workers, respectively) (Yazdi et al. 2014); Soylu et al. (215 and 42 shift and non-shift university hospital workers) (Soylu et al. 2014); Seyedmehdi et al. (406 and 309 nightshift and non-shift hospital workers, respectively) (Seyedmehdi et al. 2016); Motlagh et al. (943 professional road drivers) (Joorabbaf et al. 2017); and Aydin Guclu et al. (352 and 252 on-call shift and non-shift healthcare workers, respectively) (Aydin Guclu et al. 2019). The remaining study was a chart review (Walia et al. 2012) by Walia et al. on 884 day shift, 99 fixed evening/night shift, and 292 rotating shift workers visiting sleep clinic (Walia et al. 2012). Three studies (Aydin Guclu et al. 2019; Seyedmehdi et al. 2016; Yazdi et al. 2014) conducted assessment using the Berlin questionnaire, two studies via polysomnography (Soylu et al. 2014; Walia et al. 2012), and one study using the Stop-BANG questionnaire (Joorabbaf et al. 2017). One study (Yazdi et al. 2014) reported the ORs for males only while four studies (Aydin Guclu et al. 2019; Seyedmehdi et al. 2016; Soylu et al. 2014; Walia et al. 2012) reported sex-combined ORs. Three studies (Aydin Guclu et al. 2019; Seyedmehdi et al. 2016; Soylu et al. 2014) were conducted using HCW while the remaining two (Walia et al. 2012; Yazdi et al. 2014) studies were conducted in non-HCW.

**Table 1:** Studies included in the systematic review and meta-analysis (*N* = 6)

First author (year), country	Study design	*N*	Recruitment	Participants	Sex	Exposure variable	Outcome measures	Number of outcome events/ cases	Comparison
1. Walia (2012), USA	Retrospective chart review	1275	Clinic	Employed patients	Men and women combined	Fixed shift work and rotating shift work	PSG	day shift work: 474; fixed and rotating shift work: 216	AHI ≥ 15
2. Yazdi (2014), Iran	Cross-sectional study	470	Workplace	Workers in textile factory	Men only	Shift work	Berlin questionnaire	Non-shift workers: 204; shift workers:210	Berlin questionnaire ≥ 2 categories positive
3. Soylu (2014), Turkey	Cross-sectional study	257	Workplace	Nurses and resident doctors	Men and women combined	Shift working	PSG	Non-shift working: 1; shift working: 11	AHI ≥ 5
4. Seyedmehdi (2016), Iran	Cross-sectional study	715	Workplace	Hospital staff	Men and women combined	Night shift	Berlin questionnaire	Day shift: 25; night shift: 24	Berlin questionnaire ≥ 2 categories positive
5. Motlagh (2017), Iran	Cross-sectional study	934	Workplace	Professional drivers	Men only	Evening and night shift work	Stop BANG questionnaire	NR	Stop BANG ≥ 3 items positive
6. Guclu (2019), Turkey	Cross-sectional study	604	Workplace	Healthcare workers	Men and women combined	On-call shift	Berlin questionnaire	Day shift: 30; on-call shift: 62	Berlin questionnaire ≥ 2 categories positive

*AHI* Apnea–Hypopnea Index, *PSG* Polysomnography, *NR* not reported

### Results of individual studies

Table 2 presents the studies included for evaluating of the association between shift work and possible OSA. None of the five studies(Aydin Guclu et al. 2019; Seyedmehdi et al. 2016; Soylu et al. 2014; Walia et al. 2012; Yazdi et al. 2014) shown a significant association between shift work and possible OSA. However, a multivariate logistic regression analysis by Aydin Guclu et al. revealed a negative association of on-call shift with possible OSA (Aydin Guclu et al. 2019).

**Table 2:** Measures of the association between shift work and possible sleep apnea syndrome used in five studies

First author (year), country	Sex	Comparison	RR	95% CI (low)	95% CI (high)	Source
1. Walia (2012), USA	Men and women combined	AHI ≥ 15 vs. AHI < 15	1.03	0.92	1.15	Table 1 p.545 calculation
2. Yazdi (2014), Iran	Men only	Berlin questionnaire ≥ 2 categories positive vs. < 2 categories positive	0.86	0.44	1.67	Table 2 p.3 calculation
3. Soylu (2014), Turkey	Men and women combined	AHI ≥ 5 vs. AHI < 5	2.15	0.28	16.20	Table 4 p.108 calculation
4. Seyedmehdi (2016), Iran	Men and women combined	Berlin questionnaire ≥ 2 categories positive vs. < 2 categories positive	0.73	0.43	1.25	Table 3 p.769
5. Guclu (2019), Turkey	Men and women combined	Berlin questionnaire ≥ 2 categories positive vs. < 2 categories positive	1.48	0.99	2.22	Table 1 p.50, Table 4 p.52 calculation

*RR* risk ratio

One study performed additional or sub-group analyses. Walia et al. (Walia et al. 2012) classified shift work as fixed evening/night or rotating shift work and compared the sleep-related characteristics according to the type of work shift. Compared with day shift workers, fixed evening/night shift workers were more likely to report sleep-onset difficulties (OR 4.83, 95% CI 1.86–12.53), excessive caffeine intake (OR 3.29, 95%CI 1.19–9.12), and dozing while driving (OR 1.82, 95% CI 1.09–3.02). Contrastingly, rotating shift workers reported 2.69 times (95% CI 1.28–5.64) more difficulty with sleep onset compared to day shift workers. There was no association between work shift and apnea-related symptoms.

### Meta-analysis

**Fig. 2:**
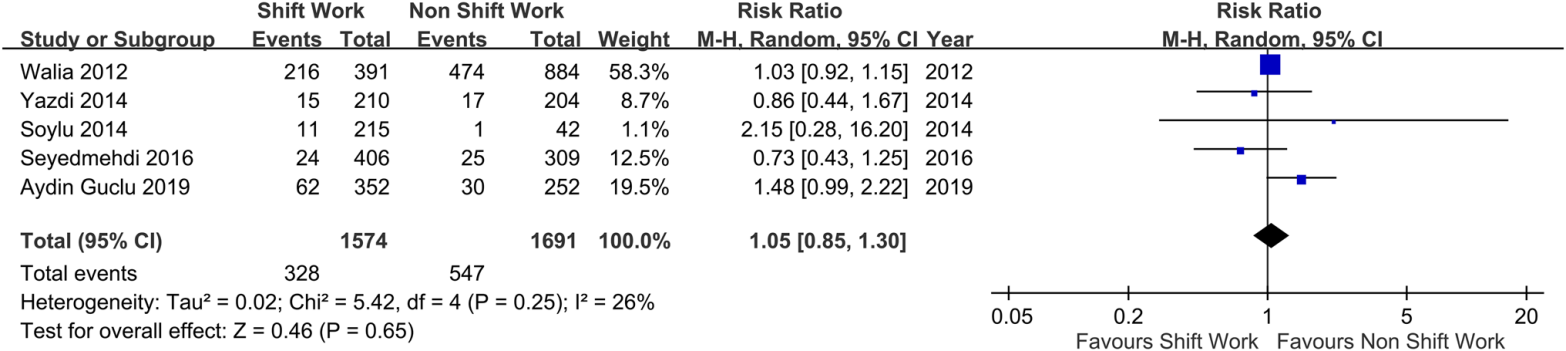
Shift work and relative risks (RRs) of possible sleep apnea syndrome in the five studies: a random-effect model

The variations in the association between shift work and possible OSA were declared by a random-effect model meta-analysis (the five studies generated RRs) (Fig. 2). The slight positive trend of the pool prevalence was not significant (RR = 1.05; 95% CI 0.85–1.30; *z* = 0.46, *p* = 0.65). The moderate heterogeneity (I2 = 26%) was also non-significant (χ2 (4) 5.42, *p* = 0.25). Moreover, the association was also slightly positive but non-significant using a fixed model meta-analysis (RR = 1.05; 95% CI 0.94–1.16; *z* = 0.84, *p* = 0.40; data available on request).

**Fig. 3:**
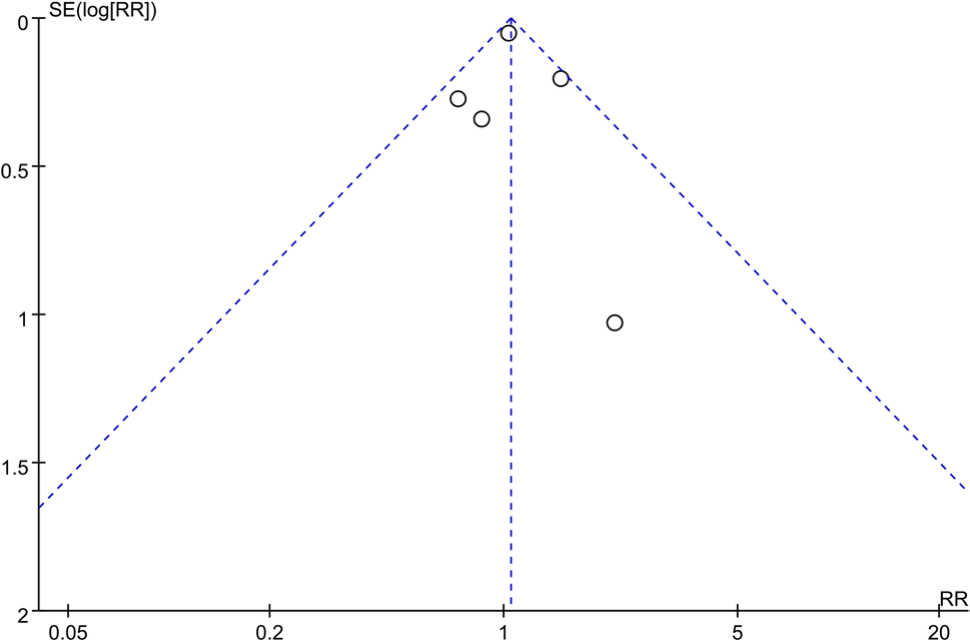
Funnel plot of log-transformed RRs of possible OSA associated with shift work and SEs for five studies

A funnel plot of the log-transformed RRs of possible OSA associated with shift work and the SEs among the five RRs revealed a relatively smaller number of studies with greater SE (i.e., smaller sample sizes) reporting greater RRs (Fig. 3). Egger's test yielded non-significant results (*p* = 0.6601).

### Subgroup analysis

We performed a subgroup analysis of HCW and non-HCW subgroups using random-effects model meta-analysis of pooled prevalence RRs (Table 3, Supplement Fig. 1). For HCW (three studies generate three RRs), the pooled prevalence relative risk was non-significant (*z* = 0.4, *p* = 0.69), with value 1.13 (95% CI 0.62–2.04). The heterogeneity was substantial but non-significant (I2 = 56%, χ2 (2) = 4.56, *p* = 0.1). For non-HCW (two studies generated two RRs) the pooled prevalence relative risk was non-significant (*z* = 0.46, *p* = 0.65), value 1.03 (95% CI 0.92–1.14). The heterogeneity was low but non-significant (I2 = 0%, χ2 (1) = 0.29, *p* = 0.59). Furthermore, the subgroup analysis of PSG and Questionnaire subgroups, male only and male-and-female combination subgroups, and different shift type subgroups shown non-significant different in shift work and non-shift work (Supplement Figs. 2, 3 and 4).

**Table 3:** Subgroup analysis of risk ratio based on whether the participants were healthcare workers

Subgroup	Risk ratio	95% Confidence interval
Study participants		
Healthcare worker group		
Soylu (2014), Turkey	2.15	0.28–16.20
Seyedmehdi (2016), Iran	0.73	0.43–1.25
Aydin Guclu (2019), Turkey	1.48	0.99–2.22
Subtotal	1.13	0.62–2.04
Non-Healthcare worker group		
Walia (2012), USA	1.03	0.92–1.15
Yazdi (2014), Iran	0.86	0.44–1.67
Subtotal	1.03	0.92–1.44

### Risk of bias assessment

Risk of bias of individual observational studies were evaluated by the risk of bias assessment tool for non-randomized studies (RoBANS). Supplement Fig. 5 revealed a low probability of bias in the except for three categories; (1) confounding variables, (2) measurement of exposure, and (3) blinding for outcome assessment. Aydin Guclu et. al., Seyedmehdi et. al., Soylu et. al., and Walia et. al. did not adjust the confounding variables. The measurement of exposure in Aydin Guclu’s and Yazdi’s studies were unclear. No attempt was made to blind outcome assessment in Aydin Guclu’s, Seyedmehdi’s and Yazdi’s studies.

## Discussion

To our knowledge, this is the first systematic and meta-analysis of the association between possible OSA and shift work. The estimated pooled prevalence RR of the association between possible OSA and shift work from the five studies was small and non-significant (pooled prevalence RR = 1.05 in the random-effects model). The present findings are not consistent with previously reported strong or moderate associations between shift work and general sleep problems (Åkerstedt 1988; Garbarino et al. 2019; Haile et al. 2019), and thus shift work may be less associated with OSA. In our study, these findings indicate that the effect of shift work on OSA remains inconclusive and may be small or negligible. They could be attributed to the varying definitions of shift work among the included studies: fixed evening/night shift, rotating shift, on-call shift, evening shift, or night shift. Furthermore, two studies (Soylu et al. 2014; Walia et al. 2012) used clinical diagnosis (polysomnography), while the other three (Aydin Guclu et al. 2019; Seyedmehdi et al. 2016; Yazdi et al. 2014) used questionnaires as the risk assessment tool of OSA. Therefore, summarizing the associations reported by the studies assessing different types of shift work and different diagnostic tool could be an inaccurate estimate in case the effects vary across the types of shift work and risk of OSA. There is a need for further future studies on specific types of shift work and consistent diagnostic tool to confirm our findings.

The small pooled association between shift work and OSA could be attributed to several confounders, including obesity and hypertension. Several studies have reported a strong association between obesity and OSA. Aydin Guclu et al. reported significant differences in the prevalence of obesity and hypertension between the sleep apnea risk and non-risk groups (Aydin Guclu et al. 2019). Soylu et al. reported that individuals with OSA had significantly higher BMI than those without OSA (31.37 ± 4.75 vs. 23.69 ± 3.42, *p* < 0.01); however, they did not assess whether BMI confounds the association between shift work and OSA (Soylu et al. 2014). Walia et al. reported that fixed evening or night shift workers were significantly more obese than day shift workers. Moreover, even after adjusting for BMI and other possible confounders, fixed shift workers were found to have 1.8 higher odds (CI 1.09–3.02) of dozing while driving compared with day shift workers (Walia et al. 2012). Further studies on the association between shift work and OAS should control for potential confounders, including obesity, hypertension, etc. Furthermore, the two Iranian studies appear to have found RRs of possible OSA was lower (Seyedmehdi et al. 2016; Yazdi et al. 2014). Yazdi et al. (2014) conducted in male workers of textile factory, while Seyedmehdi (Seyedmehdi et al. 2016) conducted in both male and female hospital workers. Compared with other studies, the relative lower RRs may be country-specific contextual factors. On the other hand, there were similar results of subgroup analysis of the association between shift work and OSA in the HCW and non-HCW groups. This suggests that different job categories might not be confounders of the association between shift work and OSA.

There have been several studies on the risk of OSA in HCW. Geiger-Brown et al. reported that 17.5% of 12-h shift working nurses had OSA (Geiger Brown et al. 2014). Soylu et al. demonstrated that 5.1% of shift workers presented OSA (Soylu et al. 2014). Seyedmehdi et al. reported a 5.9% risk of OSA among night-shift workers with a crude OR of 0.714 (crude CI 95% 0.339–1.276) (Seyedmehdi et al. 2016). Moreover, Aydin Guclu et al. reported a 17.6% risk of OSA among on-call shift workers with an OR of 0.199 (95% CI 0.053–0.747) (Aydin Guclu et al. 2019). On the other hand, among non-HCW, Walia et al. and Yazdi reported that 55.2% and 7.1% of shift workers presented OSA, respectively (Walia et al. 2012; Yazdi et al. 2014). Aforementioned studies on shift workers reported different OSA prevalence rates. This could be attributed to differences in risk factors, including age, gender, BMI, and different assessment protocols. There is a need for future studies to develop more accurate assessment protocols for determining the association between shift work and OSA in both HCW and non-HCW.

This study has several limitations. First, as aforementioned, the studies employed different OSA definitions and assessments, including questionnaires and polysomnography. There was limited research on using the diagnosed OSA and that mild symptoms may not necessarily indicate the OSA. The resulting among-study heterogeneity in the measurements may impede the interpretation of the findings and result in inaccurately estimated pooled prevalence RR. Second, the analyzed previous findings might be affected by common methodological problems with attrition bias being the most common. For example, shift workers with OSA are more likely to quit their jobs. On the other hand, the effect of healthy workers could affect the findings, since only individuals who can adapt to shift work might tolerate the working style. The lack of a clear association between shift work and OSA could be attributed to such attrition bias. Moreover, observational errors might have affected the results of individual studies. Since we did not conduct quality and methodological assessments of each study, we could not address these issues. Third, we did not consider situational factors. For example, we could not determine whether the shift work was rewarded unpaid, unwilling, or voluntary; moreover, we did not assess the shift work intensity. Fourth, we could not consider potential confounders or other occupational factors which could modify the association between shift work and OSA risk, such as obesity, hypertension, occupation, exposure to hazardous environment and substances, etc. Finally, employees with OSA might refrain from working shifts, which may affect the true association.

## Conclusion

In our study, the analysis detected no significant association between shift work and OSA (pooled prevalence relative risk [95% CI] = 1.05 [0.85–1.30], *p*=0.65). However, the available studies might be insufficient to make a clear conclusion. There was substantial heterogeneity in the methodology of the included studies. Similarly, subgroup analysis (HCW and non-HCW) indicated a non-significant association between shift work and risk of OSA. Future studies assessing more specific styles of shift work and more standardized OSA risk measures could allow more accurate findings regarding this association. In workplace practice, regular day shifts might be ineffective in reducing the OSA risk. However, future studies are required to confirm this hypothesis.

## Supplementary Information

Below is the link to the electronic supplementary material.

Supplement Figure 1. Subgroup analysis of risk ratio based on healthcare worker and non-healthcare worker

Supplement Figure 2. Subgroup analysis of risk ratio based on PSG or questionnaire (non-PSG)

Supplement Figure 3. Subgroup analysis of risk ratio based on male only and male-and-female combination

Supplement Figure 4. Subgroup analysis of risk ratio based on different types of shift work

Supplement Figure 5. Risk of bias summary

## Abbreviations

CI: Confidence interval
HCW: Healthcare worker
PRISMA: Preferred reporting items for systematic reviews and meta-analyses
RR: Relative risk
OSA: Obstructive sleep apnea
SE: Standard error
