# Supplementary figures and images for: The association between shift work and possible obstructive sleep apnea: a systematic review and meta-analysis
Source: Int Arch Occup Environ Health. 2021 Mar 7;94(8):1763–72. doi: 10.1007/s00420-021-01675-1 (PMC8490216; doi:10.1007/s00420-021-01675-1)

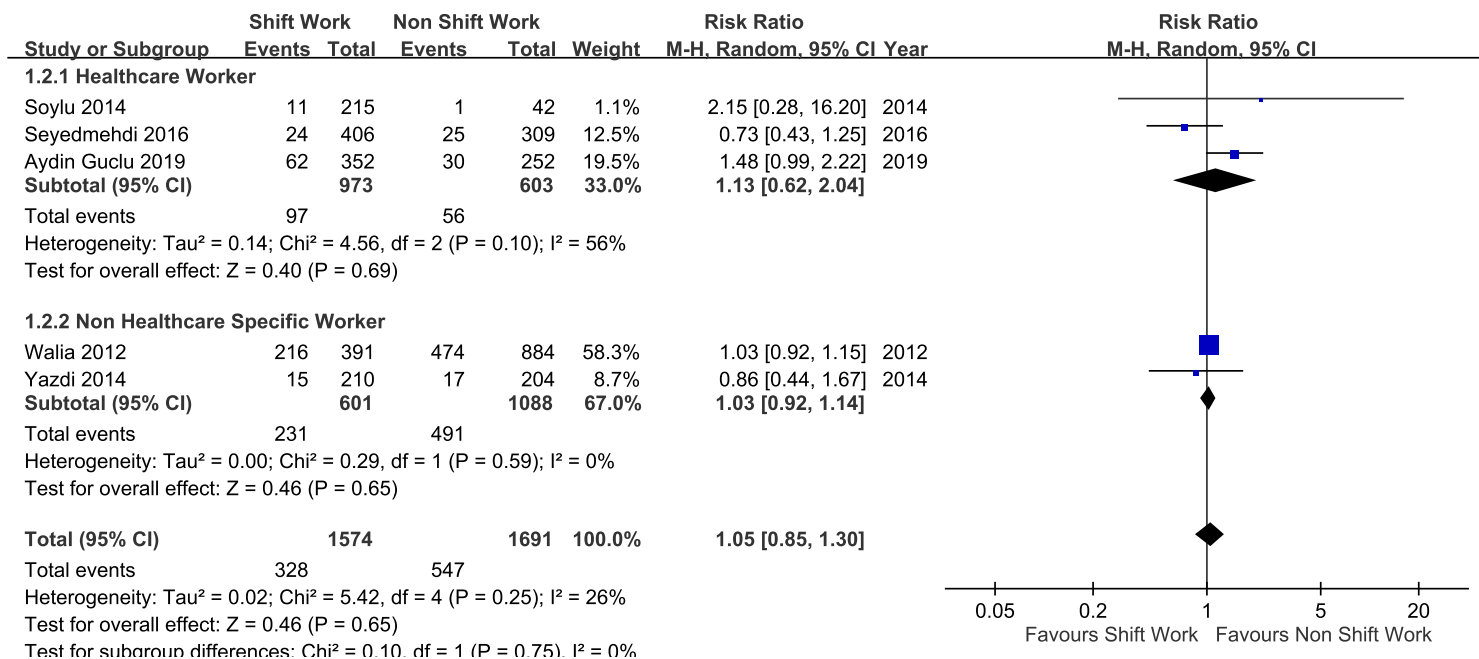

Supplement: Supplementary file 1 — Supplement Figure 1. Subgroup analysis of risk ratio based on healthcare worker and non-healthcare worker [file 420_2021_1675_MOESM1_ESM.pdf]

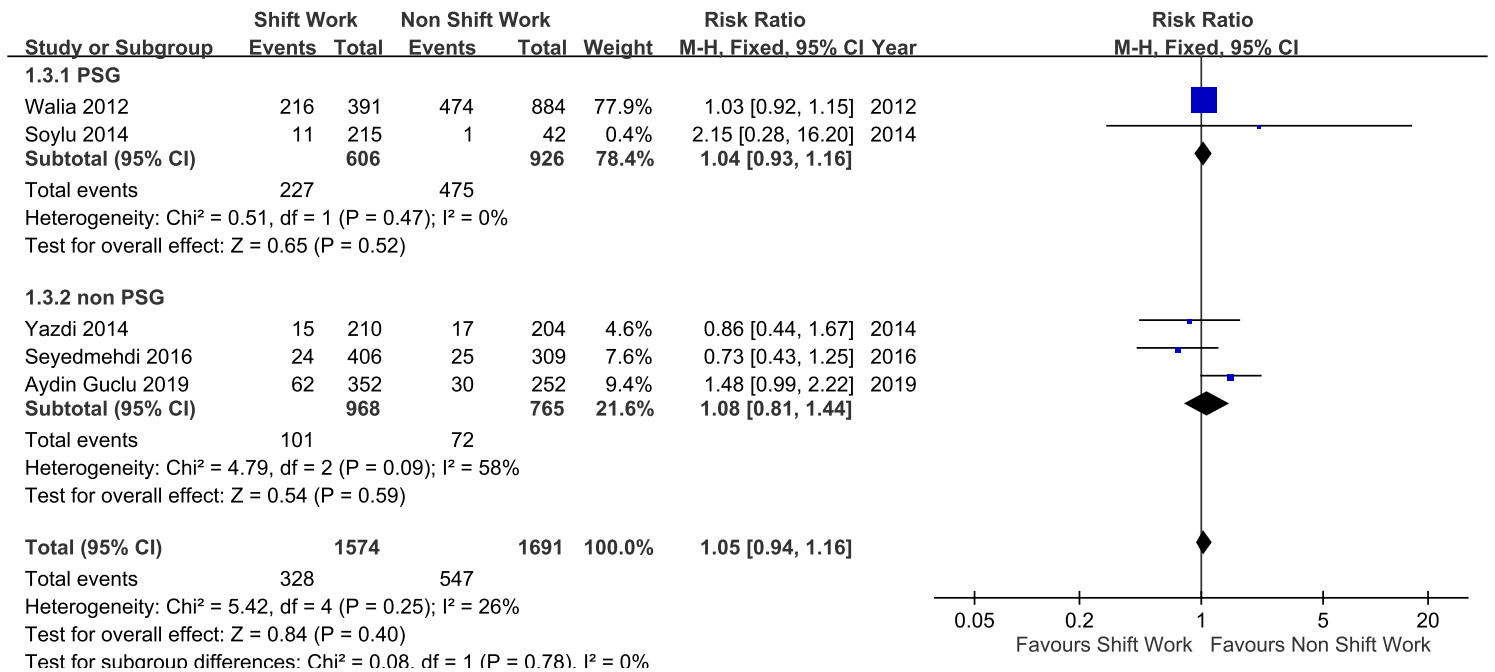

Supplement: Supplementary file 2 — Supplement Figure 2. Subgroup analysis of risk ratio based on PSG or questionnaire (non-PSG) [file 420_2021_1675_MOESM2_ESM.pdf]

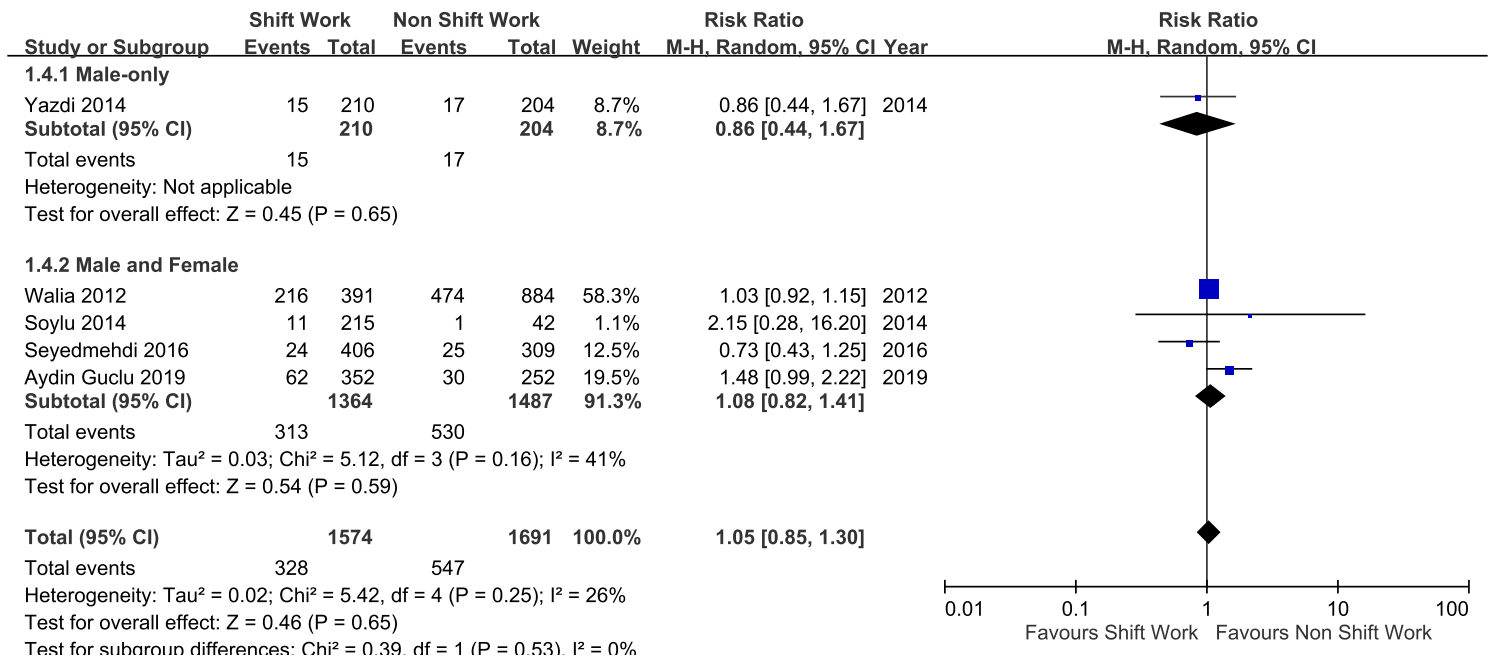

Supplement: Supplementary file 3 — Supplement Figure 3. Subgroup analysis of risk ratio based on male only and male-and-female combination [file 420_2021_1675_MOESM3_ESM.pdf]

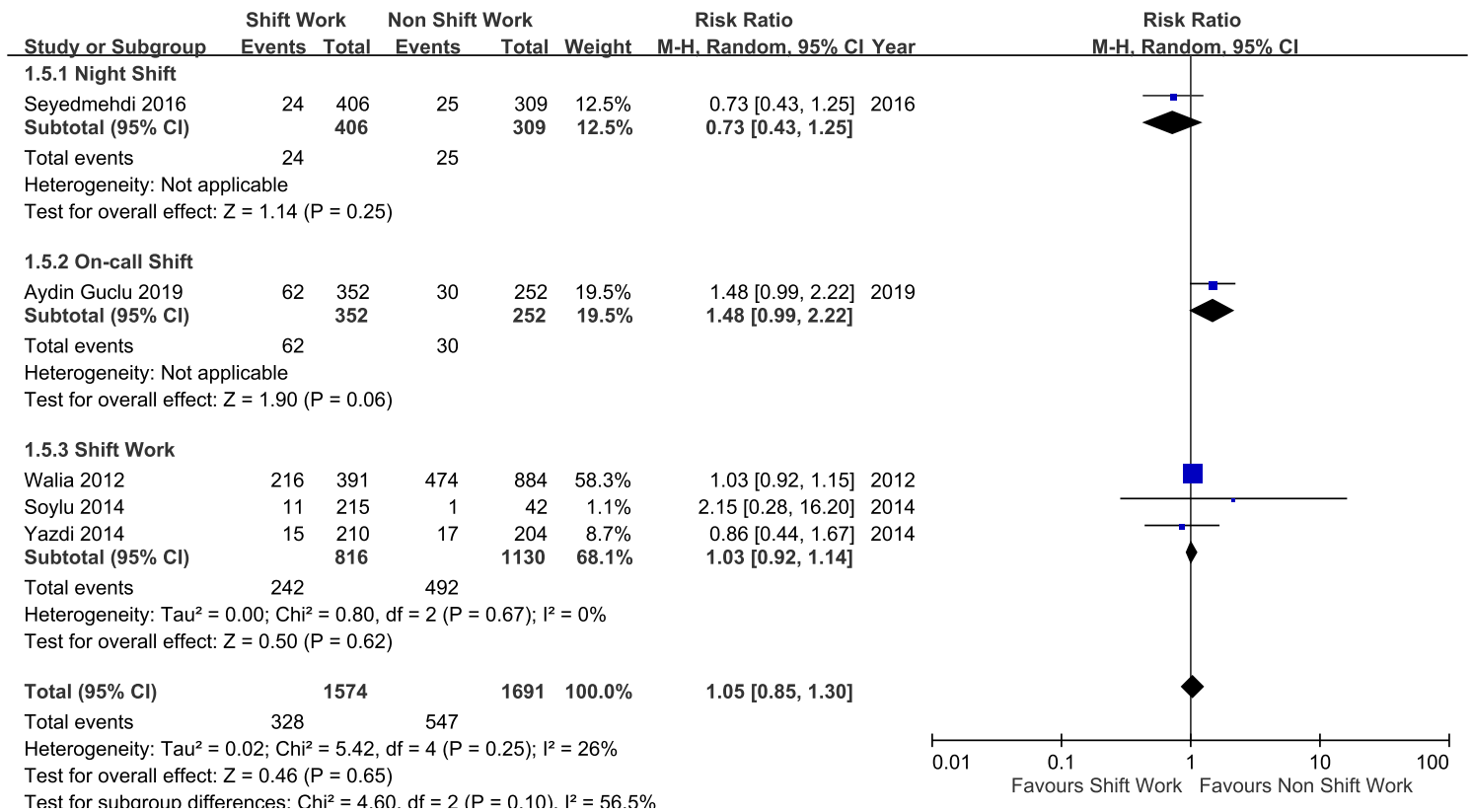

Supplement: Supplementary file 4 — Supplement Figure 4. Subgroup analysis of risk ratio based on different types of shift work [file 420_2021_1675_MOESM4_ESM.pdf]
