# Supplementary material for: The association between shift work and possible obstructive sleep apnea: a systematic review and meta-analysis
Source: Int Arch Occup Environ Health. 2021 Mar 7;94(8):1763–72. doi: 10.1007/s00420-021-01675-1 (PMC8490216; doi:10.1007/s00420-021-01675-1)
Supplement: Supplementary file 5 — Supplement Figure 5. Risk of bias summary [file 420_2021_1675_MOESM5_ESM.pdf]

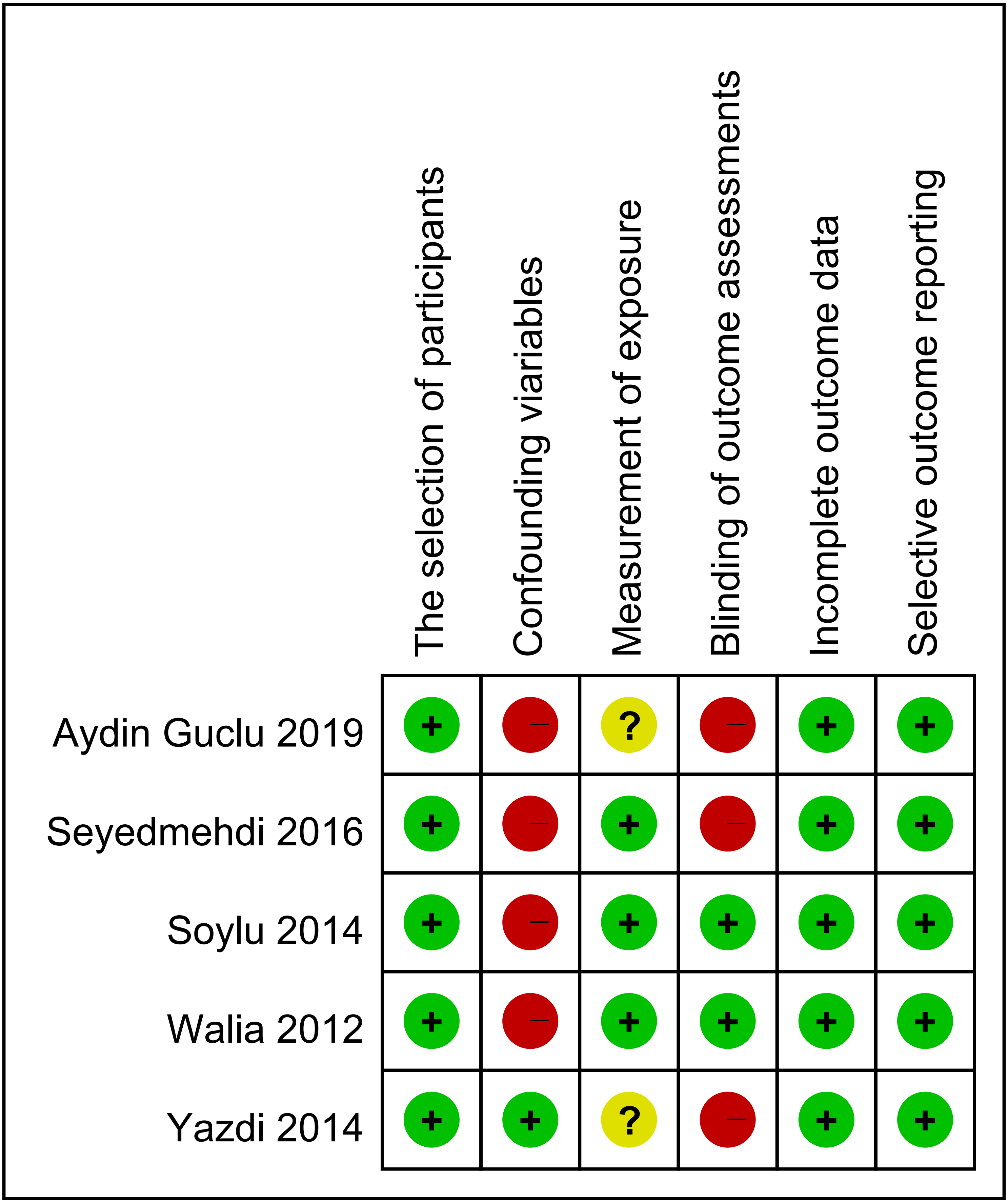
The selection of participants
Confounding viariables
Measurement of exposure
Blinding of outcome assessments
Incomplete outcome data
Selective outcome reporting
Aydin Guclu 2019
Seyedmehdi 2016
Soylu 2014
Walia 2012
Yazdi 2014
